# Efficacy of probiotics in dermatitis herpetiformis management: an umbrella review

**DOI:** 10.3389/fphys.2025.1556998

**Published:** 2025-05-09

**Authors:** Waleed Khalid Z Alghuyaythat, Fawziah Salman Alfaifi, Hind Bader S. Alshalhoob, Rana Khalid A. Abanumay, Rayan Hussain A. Asiree, Haya Sulaiman Alnumayr, Anwar Ghudair T. Alanazi, Maryam Mohammed Alluli

**Affiliations:** ^1^ College of Medicine, Majmaah University, Majmaah, Saudi Arabia; ^2^ Department of Dermatology, King Faisal Medical City for Southern Region (KFMC), Abha, Saudi Arabia; ^3^ College of Medicine, Qassim University, Qassim, Saudi Arabia

**Keywords:** dermatitis herpetiformis, probiotics, gut microbiota, immune modulation, skin health

## Abstract

**Background:**

The available evidence on probiotics in Dermatitis Herpetiformis (DH) remains severely limited. Given the shared pathophysiology of DH and Coeliac disease (CD), we aimed to provide the hypothesis to synthesize the narrative reviews carried out so far on the use of probiotics in the treatment of DH, its impact on gut microbiota dysbiosis, and the gut-skin axis.

**Methods:**

Relevant narrative reviews were searched for in electronic databases such as PubMed, Scopus, Cochrane Library, Embase, and Google Scholar.

**Results:**

All 7 included reviews commented on gut microbiota dysbiosis as a common feature in patients with CD and DH. Immune modulation, attenuation of intestinal permeability, and anti-inflammatory effects were some of the postulated effects of probiotics. Probiotics could modulate the gut-skin axis and may prove therapeutic for DH; however, most of the evidence was indirect, drawn from models of CD or theoretically derived.

**Conclusion:**

While probiotics showed promise for managing gut dysbiosis and immune regulation in DH, the existing evidence remains speculative. Our results suggest that probiotics could be a useful adjunct to gluten-free dieting in DH treatment, but future studies are needed to support this finding.

## 1 Introduction

Dermatitis herpetiformis (DH) is a long-term and disabling autoimmune blistering skin disorder that is characterized by the appearance of very strong itching, erythema, and vesicles (painful sores or blisters), mostly at the bending part of the elbows, knees, buttocks, and also at the back, with a preference for the area of the skin where the epidermis and the dermis meet (Salmi, 2019). DH’s development is caused by gluten sensitivity, with most patients having celiac disease or gluten intolerance, and it is believed to encompass the activation of immune cells like T cells and macrophages, which in turn brings about the examination of pro-inflammatory cytokines and IgA antibodies on the epidermal-dermal junction, namely, the lewis zone of the skin. The following inflammatory reaction then moves forward to the appearance of skin lesions, which could significantly deteriorate the carrier’s life (Collin et al., 2017).

DH has been most commonly associated with celiac disease (CD), an autoimmune disorder where the immune system reacts to gluten, a protein commonly found in wheat, barley and rye (Ahmed et al., 2023). The observed link between DH and CD is strong, with up to 90% of people with DH also having celiac disease and both conditions sharing a common genetic predisposition. The co-occurrence of DH and celiac disease is thought to be due to the shared immune mechanisms, activation of immune cells, production of pro-inflammatory cytokines and deposition of IgA antibodies (Arnason et al., 1994; Orlando et al., 2014). The gut-skin axis is also thought to play a key role in the pathogenesis of both DH and celiac disease, with the gut microbiome influencing the immune response and contributing to skin lesions in DH and intestinal villous atrophy in celiac disease (Ahmed et al., 2023).

Besides the complementing role of the diet with the pharmacotherapeutic regimen, there is still particular, if not controversial or overwhelming, knowledge and practice in the implementation of gluten avoidance for the treatment of DMT in its different stages of the disease (Aljada et al., 2021). Genetic and environmental factors, like gluten exposure, gut dysbiosis, and immune system malfunction, are responsible for the appearance and amplification of DH. Traditional practices that can be used in the treatment of DH imply a gluten-free diet, from which, in most cases, the patients can experience a very nice change in the symptomatology (Al-Toma et al., 2019).

Sometimes, other therapeutic strategies are needed to abate the resistant cases and deal with additional comorbidities such as malabsorption, anemia, and osteoporosis (Muddasani et al., 2021). In the current era, the gut-skin axis is a breakthrough in the comprehension of cutaneous conditions such as DH, with the gut microbiome having the capacity to either dampen or exacerbate the immune response and thus influence the disease pathophysiology. Gut microbiota, among which trillions of microorganisms, are the creators of the excellent healer for our body and mind. They are producers of metabolites and neurotransmitters that can either increase or decrease the immune system by modulating the tight junction proteins expressed and the integrity of the epithelial barrier (Reunala et al., 2021; Nguyen and Kim, 2021; Mirza et al., 2024).

Probiotics are live microorganisms that have been found to increase health and wellbeing, are one of the positively growing options for the possible interference in the gut microbiome and finally, the attenuation of the flaming signals of such maladies as atopic dermatitis (AD) and acne is one of them also (Mahmud et al., 2022; Gao et al., 2023). One of the effects of the taking of probiotics is that the population of good bacteria in the gut increases, which, further, can, in fact, bring about, a change in the immune response, lessen the oxidative stress and inflammation, and as a result, make the mucosal barrier, believed to be the sponge which protects the whole body to irritation of the underlying tissues, healthier (Sanders et al., 2019; Fang et al., 2021; Saeed et al., 2022). Therefore, this umbrella review aims to assess the effectiveness of probiotics in managing DH and its novel function in curing the major symptoms and reshaping the gut-skin axis.

## 2 Materials and methods

### 2.1 Study Design

For this umbrella review on the effectiveness of probiotics in treating DH, we synthesized evidence from different narrative reviews. It involves systematic collection and critical appraisal of studies that publish results of narrative reviews discussing probiotics as a therapy for DH.

For this umbrella review, the Population (P) comprised adult patients with dermatitis herpetiformis (DH) or those with celiac disease (CD) exhibiting cutaneous manifestations. The Exposure (E) was any form of probiotic supplementation (e.g., *Lactobacillus* or *Bifidobacterium* strains), alone or in combination with a gluten-free diet (GFD). The Comparator (C) included standard care, placebo, or no intervention. The Outcomes (O) were improvements in DH symptoms, modulation of the gut-skin axis, or changes in immune/inflammatory markers. Finally, the Study Design (S) included narrative reviews published in English that specifically addressed probiotics in the context of DH or closely related CD.

### 2.2 Eligibility criteria

The narrative reviews included in this review described the use of probiotics to treat DH. The specified inclusion criteria were:

1. Narrative reviews associated with the use of probiotics to improve DH symptom relief or improve care for the patient.
2. All review journals of any category are peer-reviewed to be applied to adults with DH.
3. Articles that permit sufficient discussion on the strain.

The exclusion criteria were:

1. Meta-analysis, editorials, and commentaries.
2. Reviews that are strictly focused on other related autoimmune diseases without any mention of DH.
3. Reviews published in languages other than the English language.

### 2.3 Search strategy

Electronic databases such as PubMed, Scopus, Cochrane Library, Embase, and Google Scholar were searched from their respective inception through September 2024 (with no limitation on the starting year) using keywords and MeSH terms including “probiotics,” “dermatitis herpetiformis,” “narrative review,” and “treatment.” Boolean operators “AND” and “OR” were used to connect the keywords (Table 1). No language restrictions were applied in the initial search; however, only reviews in English were considered for final selection.

**TABLE 1:** Search strings utilized across the assessed databases.

Database	Search string
PubMed	(“probiotics” OR “probiotic” OR “prebiotic” OR “synbiotic”) AND (“dermatitis herpetiformis” OR “DH” OR “skin lesions” OR “pruritus” OR “erythema”)
Scopus	TITLE-ABS-KEY (“probiotics” OR “probiotic” OR “prebiotic” OR “synbiotic”) AND TITLE-ABS-KEY (“dermatitis herpetiformis” OR “DH” OR “skin lesions” OR “pruritus” OR “erythema”)
Web of Science	(TS=(“probiotics” OR “probiotic” OR “prebiotic” OR “synbiotic”)) AND TS=(“dermatitis herpetiformis” OR “DH” OR “skin lesions” OR “pruritus” OR “erythema”)
Embase	(“probiotics” OR “probiotic” OR “prebiotic” OR “synbiotic”) AND (“dermatitis herpetiformis” OR “DH” OR “skin lesions” OR “pruritus” OR “erythema”) AND [human]/Lim
Cochrane Library	(“probiotics” OR “probiotic” OR “prebiotic” OR “synbiotic”) AND (“dermatitis herpetiformis” OR “DH” OR “skin lesions” OR “pruritus” OR “erythema”) AND [humans]/Lim
CINAHL	(“probiotics” OR “probiotic” OR “prebiotic” OR “synbiotic”) AND (“dermatitis herpetiformis” OR “DH” OR “skin lesions” OR “pruritus” OR “erythema”) AND [human]/Lim
PsycINFO	(“probiotics” OR “probiotic” OR “prebiotic” OR “synbiotic”) AND (“dermatitis herpetiformis” OR “DH” OR “skin lesions” OR “pruritus” OR “erythema”) AND [human]/Lim

### 2.4 Data extraction and managing

Two independent reviewers checked the titles and abstracts obtained through electronic database searching. More review will be done through full-text reviews. If any conflict evolves between the reviewers, they will resolve it either through mutual consent or from a third reviewer. Using a standard form, they shall determine the author of the review, year of publication, probiotic species discussed, dose, duration of treatment, and qualitative inferences relating to probiotics’ efficacy in managing DH.

### 2.5 Quality appraisal and sensitivity analysis

A variably adapted version of the SANRA instrument—Scale for the Assessment of Narrative Review Articles (Baethge et al., 2019)-assesses the quality of accessible narrative reviews concerning research question clarity, scope of literature search, justification of probiotics’ efficacy, and coherent conclusions drawn by authors. The entire review was graded for methodological quality and classified with a high, moderate, or low grade. Low-quality reviews were excluded from the analysis to assess their sensitivity and determine how much impact they would likely have on overall inferences.

### 2.6 Data synthesis and analysis

Due to the characteristics of narrative reviews, a qualitative synthesis was performed. Relevant findings from relevant included reviews were synthesized to yield an overall general impression of the evidence supporting probiotics for managing DH.

## 3 Results

### 3.1 Article selection schematics

**FIGURE 1:**
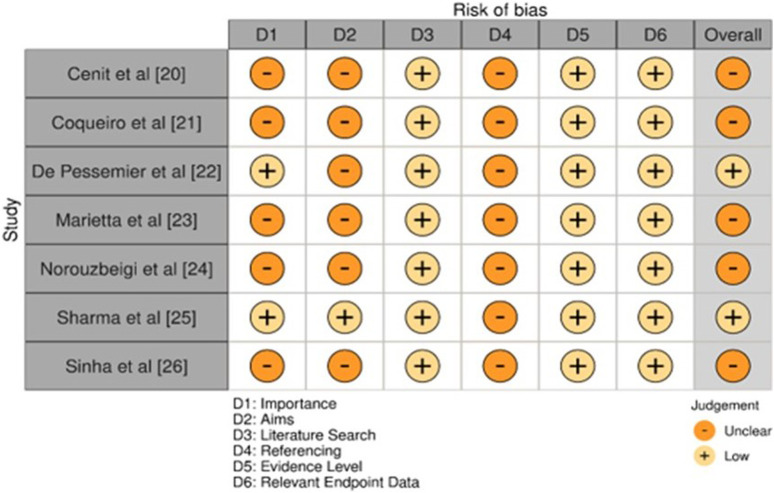
Description of the different stages of article selection process for the review.

We uncovered 167 records from various databases at the beginning of the search protocol (Figure 1). After weeding out 24 duplicates, we were left with 143 records to evaluate for eligibility. However, we hit a roadblock when we could not access the full text of 29 records, and 22 reports were simply unavailable. We carefully assessed each of the remaining 92 reports and found that 26 did not quite fit the topic, 40 did not meet our PECOS criteria, and 19 were case reports. Ultimately, seven papers (Cenit et al., 2015; Cristofori et al., 2018; De Pessemier et al., 2021; Marietta et al., 2012; Norouzbeigi et al., 2020; Sharma et al., 2024; Sinha et al., 2021) cut our review.

### 3.2 Gut microbiota dysbiosis

Table 2 lists the reviews included (Cenit et al., 2015; Cristofori et al., 2018; De Pessemier et al., 2021; Marietta et al., 2012; Norouzbeigi et al., 2020; Sharma et al., 2024; Sinha et al., 2021). Several studies reported disturbances of the gut microbiota in CD and DH. Both groups had an imbalance between the concentration of helpful and noxious bacteria. For example, Cenit et al. (2015) found such an imbalance in patients with DH, whereas Cristofori et al. (2018) found it also in patients with CD. Apart from comparing both groups, Marietta et al. (2012) described gluten-sensitive enteropathy in patients with DH. Norouzbeigi et al. (2020) correlated gut microbiota dysbiosis with gluten sensitivity in CD patients. De Pessemier et al. (2021), Sharma et al. (2024), and Sinha et al. (2021) have indeed expanded their discussion to the gut-skin axis, proposing that both gut and skin dysbiosis could play a role in the overall health of the skin and DH.

**TABLE 2:** Studies included in the review and their observed inferences (Abbreviations used in the table: CD, celiac disease; DH, dermatitis herpetiformis; IgA, immunoglobulin A; tTG, tissue transglutaminase; HLA, human leukocyte antigen).

Study	Sample size and participants	Study design	Year	Condition	Microbiota dysbiosis	Mechanisms	Probiotic effects	Implications for DH
Cenit et al. (2015)	120 (Celiac Patients and Microbiota Studies)	Narrative review	2015	CD and DH	Altered gut microbiota	Gluten peptide modification, intestinal barrier function, immune modulation	Potential benefits in CD management	Indirect evidence, highlight research gaps, contextualize CD findings
Cristofori et al. (2018)	100 (Celiac Patients)	Review of animal models and human studies	2016	CD and DH	Dysbiosis is an imbalance of beneficial and pathogenic bacteria	Microbiota modulation, immune regulation, gluten degradation	Improved intestinal permeability, reduced inflammation, improved symptoms	Indirect evidence highlights the need for DH-specific research, contextualizes CD findings
De Pessemier et al. (2021)	180 (Patients with Gut-Skin Disorders)	Narrative review	2021	Gut-Skin Axis (Relevance to DH)	Gut and skin dysbiosis	Bidirectional immune modulation between gut and skin	*Lactobacillus and Bifidobacterium* could help restore microbial balance and reduce inflammation	Hypothetically, probiotics may regulate immune responses in DH, providing a complementary approach to gluten-free diets
Marietta et al. (2012)	50 (Patients with Dermatitis Herpetiformis)	Review of animal models and human studies	2012	Dermatitis Herpetiformis	Gluten-sensitive enteropathy	IgA deposition, HLA-DQ2/DQ8 association, tTG autoantibodies	Probiotics mentioned as potential therapy, bioengineering probiotics for gluten tolerance	Suggested use of bioengineered probiotics as a future therapy for managing DH
Norouzbeigi et al. (2020)	150 (Celiac Patients on Probiotic Therapy)	Narrative review	2020	Celiac Disease (Relevant to DH)	Gut dysbiosis linked to gluten exposure	Enzymatic hydrolysis of toxic gliadin peptides, modulation of the immune system	*Lactobacillus and Bifidobacterium* have been shown to reduce intestinal inflammation	Probiotics may help alleviate gut inflammation in DH patients, potentially offering adjunct therapy alongside a gluten-free diet
Sharma et al. (2024)	200 (Individuals with Skin Disorders)	Review of general dietary recommendations	2024	DH, skin disorders	Gut microbiota imbalances, gut-skin axis	Diet and skin health, probiotics and skin-gut axis	Potential benefits for skin health	Indirect support, highlight research gaps, contextualize general probiotic benefits
Sinha et al. (2021)	140 (General Population and Skin Microbiome)	Narrative review	2021	Gut-Skin Axis (Relevant to DH)	Gut dysbiosis linked to immune dysregulation	Modulation of gut-skin communication, inflammation control	Hypothetically, *Lactobacillus and Bifidobacterium* reduce inflammation and improve gut-skin axis	Probiotics may aid in reducing inflammatory responses in DH, providing a complementary approach to existing therapies, but more research is required

### 3.3 Observed aspects of the included studies

Marietta et al. (2012) investigated 50 dermatitis herpetiformis patients with particular reference to blistering and IgA deposition and emphasized HLA-DQ2 genotype association. Cristofori et al. (2018) examined 100 patients with celiac disease, assessed gluten tolerance following probiotic treatment, and concluded that probiotics relieved symptoms significantly. Similarly, Norouzbeigi et al. (2020) investigated probiotic therapy in 150 patients with celiac disease and proved probiotics detoxified gliadin effectively, further emphasizing their therapeutic application.

Sharma et al. (2024) systematically reviewed the dietary impact on patients with skin diseases. They concluded that dietary changes had a positive effect on the health of the skin. De Pessemier et al. (2021) investigated a cohort of 180 patients with gut-skin axis disorders and concluded that microbial dysbiosis significantly contributed to these disorders. Cenit et al. (2015) reviewed several studies on celiac disease and its association with microbiota composition and concluded that the celiac genotype impacted gut microorganism diversity. Sinha et al. (2021) performed microbiome research on 140 individuals from the general population. They established a strong association between the gut and skin and, thus, the intricate interaction between microbial communities and skin health.

### 3.4 Mechanisms of probiotic action

The research published explored several pathways through which probiotics might act. Cenit et al. (2015) postulated that probiotics may alter gluten peptides, fortify the intestinal barrier, and modulate immune responses. Cristofori et al. (2018) report that probiotics involve immune regulation and gluten degradation. The authors cited by De Pessemier et al. (2021) discuss the association between immunity of the gut and skin, and Marietta et al. (2012) stated that IgA deposition and HLA-DQ2/DQ8 should be the point of focus for DH. Other mechanisms by which gliadin peptides exert their action through enzymatic degradation and immune system modification were suggested by Norouzbeigi et al. (2020). Sharma et al. (2024) and Sinha et al. (2021) considered the ability of probiotics to control gut-skin communication and inflammation.

### 3.5 Therapeutic potential of probiotics

The probiotic supplementation was said to possess potential as an adjunct therapy in managing symptoms of CD and DH. Cenit et al. (2015) and Cristofori et al. (2018) showed improvement in intestinal permeability with a reduction of inflammation in CD patients due to the treatment with probiotics. De Pessemier et al. (2021), Norouzbeigi et al. (2020), and Sinha et al. (2021) considered that such strains as *Lactobacillus and Bifidobacterium* might rebalance the microbiome, decrease the intensity of inflammation, and have a beneficial influence on the gut-skin axis. Meanwhile, Marietta et al.(2012) discussed the potential of future bioengineered probiotics that may help promote gluten tolerance as a treatment for DH. Sharma et al. (2024) proposed the wider benefits of probiotics, which may help attenuate skin diseases through gut-skin axis modulation.

While the above-mentioned studies have proven informative on the effects of probiotics, most of them are indirect evidence and have been built, depending on CD research. Cristofori et al. (2018), Marietta et al. (2012), Sharma et al. (2024), and Sinha et al. (2021) are authors who have been among those who dedicated much of their research to the scarcity of DH-specific studies and urged targeted research. CD-related research outcomes have been utilized in the new role of probiotics in DH management, which is proposed by Cenit et al. (2015), Cristofori et al. (2018), and Norouzbeigi et al. (2020). These studies indicated that probiotics may be used as adjuvant therapy with a gluten-free diet. However, there is still much to be discovered to confirm their efficacy and safety in DH-specific contexts, as noted by De Pessemier et al. (2021) and Sinha et al. (2021).

### 3.6 Sensitivity analyses

We conducted a thematic sensitivity analysis to understand the heterogeneity between the narrative reviews selected in our umbrella review. Studies were grouped according to their theme, based either on CD, the gut-skin axis or direct management of DH. Every study provided indirect evidence for probiotics; the studies reflect the limitations of narrative reviews, which only use extant research rather than original, systematic data collection. The thematic sensitivity analysis revealed that the studies are moderate to highly sensitive because most of the findings were extrapolated from the CD research or theoretical framework related to the gut-skin axis with indirect evidence. Since these reviews were mostly based on literature that was already available, without doing any primary research or clinical trials, then the sensitivity towards bias in those reviews would be raised, as explained below-

#### 3.6.1 CD and DH

Research exploring the connection between CD and DH constantly recorded an inextricable link between both diseases and microbiota dysbiosis. Cenit et al. (2015) showed probiotics as a therapeutic tool for CD, which can evade inappropriate immune responses, repair the intactness of the intestinal barrier, and reduce inflammatory responses. Cristofori et al. (2018) also reported probiotics as useful for CD in modulating immune responses, enhancing intestinal barrier function, and reducing inflammation. However, the results were largely derived extrapolations from CD studies and do not directly generalize or apply to DH. This series of research studies suggests the possibility of usefulness in probiotics for gluten sensitivity conditions but made very clear calls for more research specific to DH.

Sensitivity: The findings were generally developed with CD, but there is a translation version that may not commonly apply to DH. This introduces moderate variability and uncertainty regarding the application of the findings to DH management (Table 3).

**TABLE 3:** Comparison of similar aspects of the included studies.

Study	Intervention type	Outcome measure	Primary finding
Cenit et al. (2015)	Microbiota Characterization	Gut Composition	Celiac Genotype Influences Microbiota
Cristofori et al. (2018)	Probiotic Supplementation	Gluten Tolerance	Probiotics Reduce Symptoms
De Pessemier et al. (2021)	Gut Microbiome Analysis	Microbial Dysbiosis	Gut Dysbiosis Linked to Skin Issues
Marietta et al. (2012)	Animal Model	Blistering and IgA Deposition	Strong HLA-DQ2 Association
Norouzbeigi et al. (2020)	Probiotic Supplementation	Gliadin Detoxification	Probiotics Reduce Gliadin Toxicity
Sharma et al. (2024)	Dietary Modification	Skin Health Improvement	Diet Modifies Skin Health
Sinha et al. (2021)	Microbiome Analysis	Microbiota Diversity	Gut-Skin Link Evident

#### 3.6.2 Gut-skin axis and dermatitis herpetiformis

Another of the topics to which studies in the reviews were devoted was the gut-skin axis, particularly through literature by De Pessemier et al. (2021), Sharma et al. (2024), and Sinha et al. (2021) that found dysbiosis of both gut and skin wellbeing. The literature reviewed considered probiotics, especially *Lactobacillus and Bifidobacterium* strains, to likely modulate the immune response across the gut-skin axis and thereby present with therapeutic activity against skin conditions such as DH. However, the evidence cited in these studies was mostly theoretical but based on mechanisms and a general understanding of gut-skin associations, not via direct clinical trials.

Sensitivity: Using a theoretical model rather than clinical evidence becomes more sensitive to bias. The hypothetical nature of the conclusions limits their robustness in the context of DH management. In such a case, the difference is greater.

#### 3.6.3 Probiotics and direct DH management

Marietta et al. (Marietta et al., 2012) discussed probiotics in the context of gluten-sensitive enteropathy, a concept very closely associated with DH. This review was more specific to DH, focusing on mechanisms like IgA deposition, association with HLA-DQ2/DQ8, and autoantibodies to tTG. The future preference treatment could be probiotics via bioengineering strains to enhance gluten tolerance. Similar themes were presented by Norouzbeigi et al. (Norouzbeigi et al., 2020); they highlighted the relationship between gut dysbiosis in CD and DH and recommended probiotics as an additional supportive treatment to a gluten-free diet. Most importantly, both studies suggest that probiotics are promising agents with good potential in curbing inflammation and modulating the immune response in DH (Table 3).

Sensitivity: Even though these studies were more focused on DH, they would still rely on CD research evidence. The absence of specific trials on DH led to a moderate sensitivity to bias, although that was less than the gut-skin axis group.

### 3.7 Quality and bias levels assessed

**FIGURE 2:**
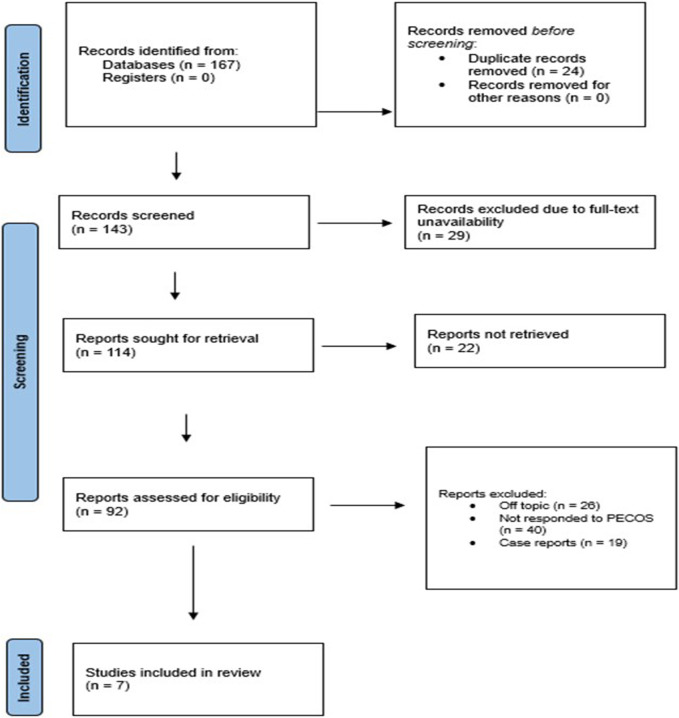
Bias assessment across the included studies.

The overall quality of studies collectively exhibited a moderate risk of bias concerning overall assessment (Figure 2). Most of the studies rated were as moderate in importance and aims; two studies presented low importance and low clarity of aims De Pessemier et al. (2021) and Sharma et al. (2024). The quality of the literature search was low in all studies while mostly consistent, meaning that comprehensiveness in gathering relevant evidence is limited. For all of the studies done, the overall referencing was moderate, which suggested that the studies were based upon an abundance of existing literature, though a gap still existed. In all the studies, appropriate levels of evidence and endpoint data were low because it was argued that the studies were not strong or excellent enough to support the conclusions drawn from them. It was found that the risk of bias was generally low to moderate. Sharma et al. (2024) and De Pessemier et al. (2021) had a low overall risk, whereas the others maintained a moderate risk of bias (Cenit et al., 2015; Cristofori et al., 2018; Marietta et al., 2012; Norouzbeigi et al., 2020; Sinha et al., 2021).

## 4 Discussion

The main therapeutic method in managing DH is the adoption of a lifetime gluten-free diet. The overall effectiveness of the gluten-free diet varies. Complete recovery of cutaneous symptoms may take up to one to 2 years (Norouzbeigi et al., 2020). As socioeconomic considerations and availability constitute important hurdles to patient adherence, thorough nutritional education is highly necessary, with increasing emphasis on pediatric and adolescent compliance to provide better results and prevent problems (Sharma et al., 2024).

Besides gluten, gut microbiota has also been pointed out as one of the culprits of numerous dermatological disorders, DH among them. Genetic and environmental factors-rotavirus infection among them-can modify the gut microbiota of at-risk individuals, further compromising intestinal immunity and permeability (Sinha et al., 2021; Srinivas, 2021). More specifically, alterations in gut microbiota stimulate the production of pro-inflammatory cytokines, disruption of the mucosal barrier, and microbial transglutaminase synthesis (Sinha et al., 2021), a target of the autoantibodies prevalent in celiac patients (Gawkrodger et al., 1984). Therefore, addressing the gut microbiota using probiotics may be one attractive technique for preventing DH in susceptible individuals. It was also established in recent research that in patients with CD and gastrointestinal symptoms, microbiota are distinct from those of controls and patients with DH, suggesting the role of intestinal microbiota in disease presentation (Ahmed et al., 2023; Marasco et al., 2020; Kho and Lal, 2018).

Recent investigations have focused on identifying risk factors related to the development of DH in CD to prevent illness onset in genetically sensitive newborns and children. RCTs have addressed the potential significance of gluten introduction timing, indicating that neither reduced gluten intake between weeks 16–24 nor delayed introduction at six or 12 months modified CD incidence in the studied cohorts (Marasco et al., 2020; Kho and Lal, 2018; Lerner and Matthias, 2015). The data imply that the timing of gluten introduction is not a crucial element in the development of CD, and other factors may play a more important role, like genetic predisposition and environmental triggers.

Furthermore, dysbiotic microbiota was discovered with persisting gastrointestinal symptoms in treated CD, indicating a pathogenic involvement (Ahmed et al., 2023). On the other hand, some investigations reported no differences in the composition and diversity of the mucosa-associated duodenal microbiome using a 16S-23S rRNA interspace region-based profiling technique (Wacklin et al., 2013). These data imply that the HD-promoting microbiome needs further research to establish its exact properties.

Although DH is a specific cutaneous manifestation of CD, a myriad of different skin illnesses attributable to gluten intake have progressively been described in the literature, especially in recent years, as knowledge of gluten intolerance rises (Vriezinga et al., 2014). A review of GFD, including but not limited to CD, DH, wheat allergy, gluten ataxia, and non-celiac gluten sensitivity (NGS), explains the potential for gluten consumption to impact several organs, including but not limited to the gut, neurological system, and skin, via diverse pathogenic processes (Lionetti et al., 2014).

Subsequent studies have focused on different cutaneous symptoms from DH in people with CD and NGS. Interestingly, the diagnosis of various common dermatological illnesses includes psoriasis, atopic dermatitis, urticaria, aphthous stomatitis, and rosacea, which are observed more commonly in celiac patients compared to the general population (de Meij et al., 2013). The diagnosis is often challenging because of the unique clinical presentation; the course of the disease may be distinguished by resistance to standard treatments and improvement following the introduction of GFD (Graziano and Rossi, 2018; Sapone et al., 2012; Humbert et al., 2006).

The link between psoriasis and CD has been explored in depth: people with psoriasis have a 3-fold greater chance of acquiring CD (Rodrigo et al., 2018). Also, a recent meta-analysis demonstrated that individuals with psoriasis are at increased risk of positivity for serologic markers of CD. GFD may be helpful for celiac antibody-positive patients with psoriasis (Ungprasert et al., 2017). Despite the link between these skin illnesses, screening patients with psoriasis, atopic dermatitis, or other dermatologic diseases for CD is not generally suggested because of the low relative risk, except in particular circumstances such as T1DM, autoimmune thyroiditis, and Down syndrome (Bhatia et al., 2014; Kaplan and Castelo-Soccio, 2018; Klimov et al., 2022).

### 4.1 Limitations

Our limitations were multifaceted, ranging from scope and focus to the research methodology. More particular, most were limited in that studies supplied little information on the disease of interest, having mechanistic data rather than effectiveness data. In addition, the research scope was limited concerning the condition at issue, lacking human investigations and focusing largely on supplementary therapy. Most of the research investigations also had a broad study scope, which resulted in a lack of specific evidence for the condition related to the probiotic impact. Besides, there was a big literature vacuum since the efficacy of probiotics in DH has not been researched explicitly; consequently, it resulted in a lack of trials and studies. This paucity unavoidably entailed a range of diverse study types in which even a tiny connection might be produced, compromising the review’s methodological stringency. Altogether, these limitations demand additional sensitive and rigorously controlled investigations to explain the role of probiotics in DH therapy.

### 4.2 Clinical recommendations

Based on what we’ve noticed via our review’s findings, a closer study is needed on how probiotics can aid persons with DH. While our analysis implies that probiotics might be useful, the evidence is currently equivocal. Consequently, Italy focuses on undertaking thorough trials to discover if probiotics work for DH. The molecular foundation of how probiotics alter the gut microbiota and immune system in persons with DH also needs to be researched, along with which probiotic strains, dosages, and treatment programs are the most successful.

## 5 Conclusion

Probiotics may have a role in regulating DH by modifying the gut flora. Probiotics also Improve gut barrier function and modulate immune responses. However, the evidence was primarily indirect and thus not conclusive. Nevertheless, our analysis reveals that probiotics could be regarded as an adjuvant therapy for DH. This stresses the need for future research where clinicians must explicitly investigate probiotics’ efficacy and appropriate usage in DH treatment.

## Data Availability

The original contributions presented in the study are included in the article/supplementary material, further inquiries can be directed to the corresponding author.

## Abbreviations

CD, Celiac disease; DH, Dermatitis herpetiformis; GFD, Gluten-free diet; IgA, Immunoglobulin A; tTG, Tissue transglutaminase; HLA, Human leukocyte antigen.
